# Pavement Texture–Friction Relationship Establishment via Image Analysis Methods

**DOI:** 10.3390/ma15030846

**Published:** 2022-01-23

**Authors:** Ivana Pranjić, Aleksandra Deluka-Tibljaš

**Affiliations:** Faculty of Civil Engineering, University of Rijeka, 51000 Rijeka, Croatia; aleksandra.deluka@gradri.uniri.hr

**Keywords:** pavement texture, image analysis methods, skid resistance, digital texture model

## Abstract

Pavement surface texture is one of the prevailing factors for friction realization on pavement surfaces. In this paper, an overview of pavement texture properties related to the pavement frictional response is given. Image analysis methods used for pavement texture characterization are thoroughly analyzed together with their potential for the establishment of a pavement texture–friction relationship. Digital pavement surface models derived from photogrammetry or laser scanning methods enable the extraction of texture parameters comparable to the ones acquired by common pavement surface measuring techniques. This paper shows the results of a preliminary small-scale research study of the pavement texture–friction relationship. This research was performed in a laboratory which produced asphalt samples, primarily to analyze the potential of developing a methodology for the digital pavement texture model setup. Furthermore, the relationship between selected 2D texture parameters calculated from the digital texture model and measured friction coefficient expressed as SRT value was analyzed. A significant correlation was established for standard texture indicator mean profile depth (MPD) and SRT values (R = 0.81). Other texture parameters showed moderate correlation with the frictional response of the surface, with absolute values of correlation coefficients varying from 0.7 to 0.75. A further analysis of this relationship will be performed by inclusion of other texture parameters that can be determined from the digital texture model acquired by the established methodology.

## 1. Introduction

Friction force acts as a resistance to the relative motion of a body moving over a nominally rough substrate along the contact area of the bodies in interaction. The force that is developed when a partially or fully blocked tire slides over a pavement surface is called skid resistance, and it is a result of the frictional properties of a pavement [1]. The friction coefficient directly affects the stopping sight distance and vehicle stability in curves [2]; thus, it is not an exaggeration to state that it is one of the most important properties in road safety management. One of the influencing factors for the friction realization which can be analyzed and monitored by pavement engineers is pavement surface texture.

Pavement texture represents a deviation of the pavement surface from an ideal plane within a specific wavelength and amplitude range [3]. The limit values of wavelength and amplitude range define different texture levels: micro-texture, macro-texture, mega-texture and unevenness. The influence of each of the texture levels on pavement surface performance is different: micro-texture affects friction at low speeds, macro-texture has a significant effect on high-speed friction, rolling resistance, surface water drainage and noise, while mega-texture and unevenness mostly govern riding comfort and vehicle wear [2]. Despite numerous investigations having been performed in order to establish the texture–friction relationship, there is still no straightforward relationship established between the pavement texture properties and the friction occurring on the vehicle–pavement contact. According to [4], this might be due to the complexity of the mechanism which cannot be described by just one texture parameter, or by analyzing only one influencing factor; for example, speed or water film thickness on the contact surface. Pavement engineering research interest for the friction realization is mostly concentrated on the pavement texture properties, investigating either the influence of different texture levels separately [5,6], or emphasizing the importance of the combined effect of both texture levels on the skid resistance [7]. Even though the PIARC experiment [2] derived a harmonization procedure for the texture and friction measurements from different measuring devices through the established international friction index (IFI), the results are limited just to the measuring output values, and not the phenomenon itself. Previously performed research points out that traditionally measured higher texture values do not always yield higher measured friction values which can be related to the limitations of the standard measurement methods with the resulting texture parameters and their influence on the friction [7,8,9,10].

Previous research of the texture–friction relationship reported in [11] was performed on large sets of macro-texture and friction data collected in situ, by means of standard measuring methods. Data analysis showed a weak correlation between the two inspected parameters. These results motivated further research of the texture influence on pavement frictional performance by analyzing pavement texture in terms of image analysis methods. Image analysis methods represent an alternative way of pavement texture data acquisition, where texture parameters related to the frictional response of the inspected surface are not provided as a measurement result, but they have to be calculated from acquired images data. Research performed in the last decade in this field is focused on defining advanced methods for data acquisition and texture properties determination. In general, data acquisition concerns two different devices—a digital camera or a laser scanner, from which acquired data are mostly reconstructed in a 3D surface model, which is further analyzed by means of texture-related parameters that could contribute to the understanding of the frictional response of the surface.

This paper is a result of a small-scale research study designed to explore the possibility of using image analysis methods for the description of the pavement texture and friction relationship in a simple and efficient way. Prior to the experimental phase of the study, a detailed overview of recently performed research exploiting advanced methods for pavement texture determination based on digital image analysis was performed. The main objective of this research was to establish a new simple and reliable methodology for 3D digital pavement surface model setup by using selected image analysis techniques. The methodology is based on the photometric data acquisition technique by using a standard digital camera and further data analysis with one commercial and one open-source software. By applying orthographic photogrammetry technique for data acquisition, the asphalt slab surface was digitalized and further analyzed by means of texture parameters that can be calculated from the dense point cloud data. Calculated texture parameters were correlated to the measured friction coefficient of selected asphalt slab sections, in order to inspect if texture data obtained from the digital pavement surface model could indicate the frictional performance of the inspected surface.

## 2. State-of-the-Art of Pavement Texture Characterization via Image Analysis Methods

The pavement surface is an irregular surface determined by randomly distributed asperities, formed as a result of asphalt mixture properties and the pavement surface layer placement method. According to the PIARC categorization [2], texture levels responsible for the surface water drainage, the friction coefficient and the vehicle tire wear are micro-texture and macro-texture. Micro-texture is defined as surface texture with wavelengths shorter than 0.5 mm and amplitudes lower than 0.5 mm, resulting mostly from mineral properties of the aggregate in the asphalt mixture [6]. Harder stones such as granite produce higher levels of micro-texture, while stones such as limestone produce lower micro-texture levels. The most important aggregate properties in relation to the micro-texture listed in the research by [12] are geometric characteristics—shape and size, and petrological and physical properties, mostly resistance to polishing. When inspecting pavement texture characteristics on low-speed roads, the values of micro-texture are usually more emphasized and related to the available skid resistance level. However, as stated in [1], micro-texture is fundamentally important for both wet and dry pavements and relevant for the frictional properties, regardless of the driving speed. Micro-texture is related to the micro-roughness of the pavement surface, so it is difficult to measure it directly on site. Therefore, it is usually described by the results from the low-speed skid resistance measurements, such as the pendulum tester, where the measuring result is expressed as skid number or friction coefficient [13]. In this way, however, micro-texture is not accurately determined, and its influence on pavement frictional performance cannot be unambiguous, but includes the effect of macro-texture and other influencing factors during the measurement.

Macro-texture represents a range of surface textures with wavelengths between 0.5 mm and 50 mm and amplitudes between 0.5 mm and 20 mm [2]. It is a result of asphalt mixture properties and placement method, and can be described as the texture of the road surface visible to the naked eye. The values of macro-texture depend on several influencing factors, where the aggregate mineralogy, distribution and gradation in the mixture, binder type and content and the air voids volume are detected as the most significant, as stated in [6]. Macro-texture values are relevant for high-speed roads, since they have a more significant impact on skid resistance in such conditions, together with the ability to drain the surface water and reduce the thickness of the water film.

The determination of standard macro-texture parameters is an established procedure defined in regulations [14]. The methods for macro-texture determination are different according to the measuring principle, and categorized as contact and non-contact measurements with output parameters, as shown in Table 1. The standard macro-texture parameters as MTD or MPD give a general representation of macro-texture properties and performance. However, they cannot be used to exactly describe the relationship between texture and friction on pavement surfaces. Therefore, advanced methods for pavement texture characterization have recently been in development, in order to overcome the limitations of standard texture characterization methods, and provide better insight into the texture–friction relationship establishment.

Image analysis methods are in development for a broader application in various engineering fields, allowing the non-contact measurement of inspected entities, and a very realistic 3D perspective of the measured objects. The general principle used in all image analysis methods is to acquire high-precision digital representations of an object, and use it to extract significant properties relevant for the research objective [15,16]. Photometric technologies enable simple data acquisition and the monitoring of structure deformation, either by single- or multi-vision reconstruction techniques for problems concerning surface and structure deformations, measurement of stress and strain fields, structure and surface reconstruction, moving object reconstruction, and others [17,18]. Image analysis methods enable the description of 2D and 3D features of pavement surfaces responsible for friction development. This possibility is one of the significant advances, in comparison to the traditional texture characterization methods, where micro-texture values cannot be determined directly, and macro-texture is described just as a profile-based parameter.

One of the methods for texture data acquisition is by using a single or multiple digital cameras, either as a structure-from-motion photometric stereo system with multiple images of an object captured from different angles, or as a photogrammetry method with a moving camera positioned orthogonally, or tilted in relation to the object’s surface [1]. Another technique used for digital image acquisition is the 3D laser scanning, but less common due to the necessary equipment, which is more expensive than the digital camera. Each of these methods results in a set of data relevant for further image analysis in terms of pavement texture characterization. Table 2 shows a summary of image analysis methods with the resulting entities and most common calculated texture parameters. The characteristic advantages and limitations for each method are listed in comparison to standard or other advanced methods.

The output parameters resulting from image processing are texture descriptors that can be compared or related to the frictional response of the analyzed pavement surface. Image analysis methods provide several groups of surface topography parameters significant for the texture characterization. They are usually divided into 2D or profile-based parameters and 3D or spatial parameters, analyzed by means of statistical or geometrical values [7]. Profile-based parameters are used to characterize the texture properties along the inspected profile, while spatial parameters are used to describe the texture properties on the inspected surface. With image analysis methods, both significant texture levels (micro- and macro-texture) can be acquired and further analyzed in terms of profile or spatial-based characteristics, which is one of important advances in comparison to the traditional methods, where micro-texture values cannot be determined directly, and macro-texture is described just as a profile-based parameter.

Profile-based parameters are derived from summits or peaks of the inspected texture profile, and they are usually statistical representations of the surface properties [4]. This type of parameter is the most common for pavement texture characterization, and some of them can be determined by standard texture measuring methods, for example MPD. Other profile-based parameters that can be extracted by image analysis methods are peak radius, peak height, and peak curvature [3].

Spatial parameters are investigated in order to better describe and understand the texture and friction relationship on the contact surface between the pavement and the vehicle tire [5,10,24]. They are usually grouped in height or amplitude parameters, spacing parameters, hybrid parameters, and functional or feature parameters [37]. Height parameters are related to the statistical distribution of height values, spacing parameters involve the spatial shape of the surface data, hybrid parameters are a combination of the previous two, and functional parameters represent the surface structure related to the material behavior properties, and they play a very important role in the determination of the pavement surface skid resistance, but also for the statistical analysis of the macro-texture and friction relationship. Spatial parameters can be obtained from both 2D and 3D surface representations, depending on the type of data acquisition—profilometric or areal analysis of the surface profile. Several researchers have investigated the differences between the 2D and 3D parameters, resulting in expressions for the calculation of the spatial parameters from the spectral moments [38]. The spectral moments m^0^, m^2^ and m^4^ previously calculated from the 2D profile measurements are the mean square height, root mean square slope square, and second derivative of the profile, respectively. The spectral analysis is incorporated, since the spectral parameters are shown to be scale-independent, due to their estimation along multi-scale measures, which enables a wide range of texture wavelengths to be included in a single analysis [4].

Texture parameters can also be classified as geometrical or statistical classes, which can both be analyzed by 2D or 3D frameworks [16]. The geometrical class of indicators involves average roughness, peak to valley height, leveling depth, surface roughness depth and they are a result of surface texture characterization by using the profilometers. The statistical class of indicators involves the variance, average quadratic deviation, skewness, and kurtosis. All the 2D indicators can be extended to the 3D parameters for texture characterization by using defined mathematical expressions, thus enabling the possibility of volumetric analysis, which is of particular interest for hydroplaning effects related to the macro-texture level.

Image processing methods can be categorized as low, intermediate, and high processing levels, different for its output results, which can be images, image attributes, or properties and functionalities of the objects in analyzed images [20]. For example, texture is a characteristic which can be related to the physical property of an image-captured surface, and provide information about the structural arrangement of the surface. When a surface image is analyzed, the results of image processing can provide information about the regions in the image which have the same reflectance or luminous difference; namely, the spatial frequency, which can be further analyzed using mathematical algorithms. One of the most employed techniques is the analysis of texture image in the frequency domain with power spectral energy [25,27], where a rough texture has a large spectral period and low frequency concentrated spectral energy. Investigations of pavement surface digital images in terms of fractal dimensions (area and contour) are shown in [36,39], correlating these parameters to standard representations of surface roughness and friction. Research done by [40,41] explains how an analysis of macro-texture properties in terms of frictional properties is performed through wavelet analysis, which involves discrete wavelet transformation. Acquired surface texture data are decomposed in various wavelengths and analyzed using wavelet energy indicators.

In general, image analysis methods provide more accurate and detailed information about the pavement texture properties in comparison to the standard texture characterization methods. This is especially emphasized with the 3D techniques, where important spatial parameters significant for the understanding of the pavement texture and friction relationship can be extracted [30,31]. The main disadvantage of the photometric methods is that several types of errors in the data acquisition procedure can influence the accuracy of the data. The most common errors listed in research performed by [23,42] are the presence of specular reflectance or shadows, deviations in the light source orientation or mismatched irradiance, imaging geometry, factors related to the surface material, and others. The texture data acquisition by 3D laser scanners is faster and less attributed to some of the errors listed above regarding photogrammetry methods. However, the 3D scanners are more complicated to use and more expensive than digital cameras which are used in photometric techniques [1]. Therefore, they are less commonly used, despite the disadvantages of the photometric methods in comparison to 3D laser technology. In general, the procedure of image processing and the interpretation of the results within a 3D point cloud data or mesh in terms of texture parameters identification are more complex and time-consuming than the traditional methods for texture parameters determination.

## 3. Materials and Methods

A small-scale research study was performed in order to establish the methodology for generating a digital pavement texture model by using selected image acquisition and analysis techniques. The goal of the preliminary research was to generate a reliable digital pavement surface model, so that the characteristic texture parameters can be determined and used for the texture–friction relationship analysis. The experiment was performed on a laboratory-produced asphalt sample with characteristic properties described further in the text.

The investigation was carried out in two phases:

- Phase 1, where the frictional properties of the sample were investigated;
- Phase 2, where the sample was transferred to a 3D surface model used for further pavement texture characterization.

### 3.1. Sample Preparation and Measurement of Frictional Performance

In this experiment, a standard hot asphalt mixture wearing course was selected for the determination of texture properties related to the pavement surface frictional performance. The selected asphalt mixture type is commonly used for heavy trafficked roads in local conditions, such as highways or urban high-speed roads. These types of roads are usually subjected to macro-texture inspection and monitoring by using standard measuring devices, such as vehicle-installed profilometers, with output data interpreted as MPD or ETD value. Collected macro-texture data are afterwards used for the evaluation of frictional performance on measured roads.

**Figure 1:**
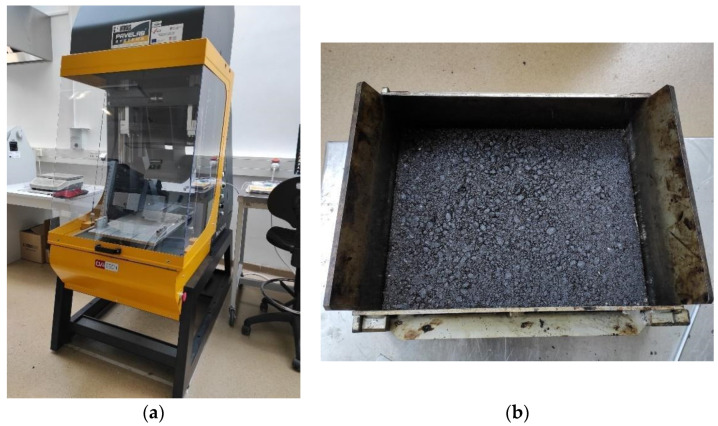
Roller compactor (**a**) and the produced asphalt slab (**b**).

The selected mixture is categorized as AC 11 surf BIT 50/70: eruptive aggregate type from a local quarry with nominal maximum aggregate size 11 mm, and paving grade bitumen with penetration grade 50/70. This mixture was used for the production of a rectangular slab (Figure 1b) dimensions 400 mm × 300 mm × 40 mm with a laboratory roller compaction device (Figure 1a). The dimensions of the asphalt slab are selected to be suitable for the further inspection of frictional properties and the analysis of texture characteristics.

**Figure 2:**
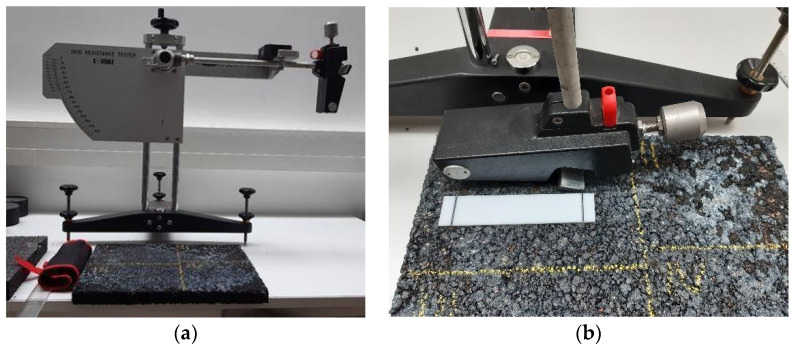
SRT device in the experiment setup (**a**) and close-up view of measurement setup (**b**).

In Phase 1 of the experiment, the produced asphalt slab was inspected in terms of frictional performance by using the skid resistance tester device SRT (Figure 2). This device operates on a pendulum principle, where the pendulum arm is released from the initial horizontal position and falls freely until the rubber pad mounted on the pendulum head touches the inspected surface (Figure 2a). The surface roughness provides the resistance to the further motion of the pendulum arm, and the amount of the resistance is characterized as a skid resistance number that can be interpreted as the coefficient of friction [43]. This device simulates the anti-skid performance of the road surface for low-speed friction, and it is used both for laboratory and in situ analyses of the frictional performance of pavements [13,44].

The asphalt slab was divided into 4 sections, where the friction coefficient expressed in SRT value was determined following the procedure defined in relevant technical regulation (Figure 2b) [13]. The sections were formed as four rectangles so that the central parts of the slab can be inspected during the SRT measurement procedure. The dimensions of the sections were selected with respect to the necessary length and width for the SRT measuring device. In each section, the testing area of approximately 130 mm long and 80 mm wide was selected in the central position. The dimensions correspond to the sliding distance and the width of the pendulum rubber during the test. The friction measurement was performed on each section as an average of five readings of the SRT value, in conformity with the procedure described in the relevant standard [13]. The measurements were performed on dry surfaces in order to exclude the effect of the water film on the testing results.

### 3.2. 3D Texture Model Generation by Image Analysis Methods

**Figure 3:**
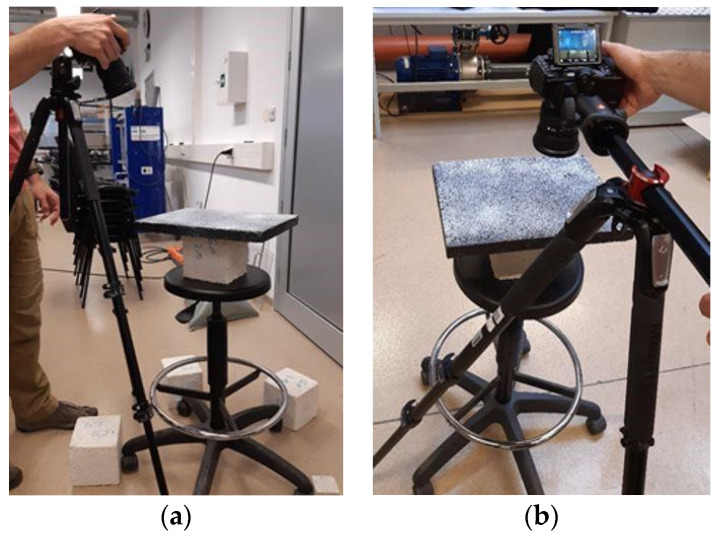
Image acquisition methods: structure from motion (**a**) and orthographic photogrammetry (**b**).

Phase 2 of the experiment was oriented on the creation of a digital texture model from the produced asphalt slab by means of image analysis methods. The selected data acquisition method was photometric, due to its availability and previous experience in its application to larger scale problems, such as coastal erosion monitoring and scaled landslide monitoring by photogrammetry methods [45,46,47]. The methodology exploited for the digitalization of the geometry of larger scale problems was modified and adjusted to be applicable to a small-scale problem analyzed in this research. A similar methodology was used for road surface reconstruction and analysis in terms of structural behavior and road surface defects detection on roads in use [48]. In this research, pavement texture data were collected by two different photometric methods in order to test their applicability for generating the digital model of the pavement surface. Prior to the data acquisition, asphalt slab was cleaned from the markings from Phase 1 and sprayed with an anti-reflectance spray to reduce the influence of the ambient light and minimize the possible errors during the image acquisition method. The slab was positioned on a turntable with a stand lifting the slab from the table surface to reduce the influence of the contact between two surfaces in the image processing procedure. The entire assembly is rotatable for 360 degrees, but it can also be in a fixed position. The image acquisition process was performed by structure from motion (SfM) and orthographic photogrammetry methods (Figure 3). The photographic equipment used in both methods was digital camera Nikon D500, 50 mm, F1.8. Prior to the data acquisition, the camera settings were adjusted to have equal brightness, contrast, and sharpness properties for both of the photometric methods applied. In this way, the quality of acquired images was the same for both exploited methods. The acquired images resolution was 20 megapixels for both applied methods.

In the SfM method, the camera was fixed on a mount, and the slab was rotated 360°. The photos were acquired with 25° shift at three different heights, where, at each height, approximately 15 photos of the whole slab surface were taken. The number of photos was determined according to the texture characteristics in a specific position in the slab. For example, in the areas where the slab was more textured, more photos were captured in order to be able to describe texture more accurately later in the model. Overall, 45 photos were taken using the SfM method. In the orthographic photogrammetry method, the slab was fixed, and the camera was moving perpendicular to the slab area along the longer edge, capturing overlapping section photos of the slab. Additional photos of the sample were taken along all four slab edges with a camera under 45° in order to gain a better insight of the texture depth. Overall, 30 photos were taken using the orthographic photogrammetry method. For both methods, the images were captured in RAW format, giving the real information from the camera sensors. In this way, no pixel data were lost, and acquired images are not interpreted or pre-processed, which is important for further data analysis. Images in RAW format are adjusted in a way that the brightness and contrast settings are optimized to obtain the best possible image quality for further data analysis. Special attention was paid to the sharpness of the images by defining a high contrast, and consequently acquiring images with correctly exposed pixels. Later on, images are saved in an incompressible image tiff format, containing the data for each captured pixel. The pixel size on each captured image was 4.45 × 4.45 µm, so both macrotexture and some of the microtexture features were captured with the equipment used.

The slab photos in tiff format were inputted in a commercial image processing software Agisoft Metashape by Agisoft LLC, St.Petersburg, Russia. The software is used for the construction of the point cloud or mesh surface 3D model from the acquired digital images. The surface model reconstruction begins with an image alignment procedure. In order to generate as realistic a surface model as possible, approximately 40 thousand points from each captured image are analyzed and compared with each other in order to extract the points with common properties, and use them for further reconstruction. The alignment procedure results in approximately 5000 selected points in each image, which are the best for surface representation. Afterwards, the images defined by selected points are additionally filtered by using reprojection error, reconstruction uncertainty, and projection accuracy filters. In this way, the points that do not fall within the defined error threshold are dismissed, and the accuracy of the model is further improved. The points acquired after the alignment and filtering procedure represent a basis for 3D surface model creation. An alignment and filtering procedure was performed for both data acquisition methods. Since both methods resulted in a similar set of points, the orthographic photogrammetry method was selected for further analysis. The main advantage of this data acquisition method in comparison to the SfM method is the shorter duration of the alignment and filtering procedure before the model creation, because fewer images were taken using this method. Another reason is that the alignment procedure was slightly simpler because the data comparison was made for slab surface segments images, and not for the entire slab at once, which also reduced the processing time. Besides the benefits in the image processing aspect, the orthographic photogrammetry method is much more suitable for surface model representation when considering the possibility of implementing the developed methodology for in situ measurement and the monitoring of constructed roads. By using one or more cameras positioned orthogonally to the pavement surface and installed on a vehicle-driven system, the monitoring process could be done by applying the methodology of image acquisition presented within this small-scale study.

The final set of points defining captured images was further exploited for the generation of a 3D dense point cloud model. As the resulting points represent the best fit to the actual surface, they were used in an interpolation procedure for generating additional points for surface representation. In the first iteration, approximately 6.5 million points were generated. These points were further filtered according to the measurement uncertainty, with threshold values corresponding to the upper and lower limit values of relevant pavement surface texture scales micro-texture and macro-texture. The result was a dense point cloud with nearly 6 million points, which was used to create a 3D mesh model of pavement surface defined by points coordinates XYZ. The generated 3D asphalt slab mesh model was imported into an open-source software suitable for dense point cloud (DPC) analysis *Cloud Compare*, and subjected to analysis of texture characteristics related to the frictional performance.

The initial analysis step consisted of the *Cloud Compare* DPC creation from the 3D mesh model generated in the Agisoft Metashape software, in order to describe the slab texture as a set of XYZ coordinates. In this way, the texture is stored as a 3D entity defined with coordinates, and it can be used for further analysis in any software which accepts datasets stored in this way. The DPC generated from the trial sample consisted of approximately 10^6^ points. After the initial data preprocessing, scaling and leveling of the DPC model, it was divided into four sections equal to those defined for the SRT measurement. Each of these sections were further analyzed by extracting the sub-sections where the SRT measurements were taken. The sub-sections were extracted as separate point clouds with dimensions approximately equal to the sliding area of the pendulum rubber (120 mm × 75 mm). From each of the extracted sections, several 2D profiles were obtained in order to calculate the average mean profile depth (MPD) of the surface, and compare it to the measured SRT values. The established methodology for 3D texture model generation and preparation for the texture data analysis is summarized in Table 3.

## 4. Results and Discussion

**Figure 4:**
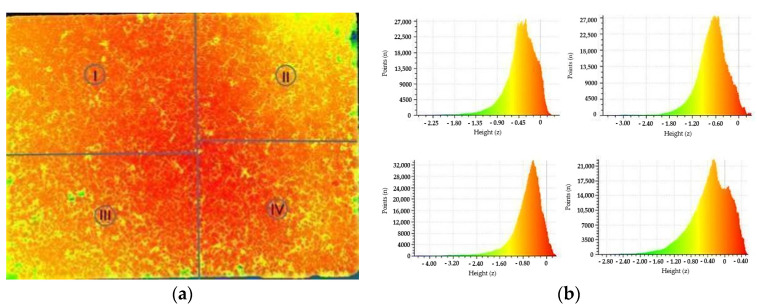
Height maps of analyzed slab sub-sections (**a**) and corresponding histograms (**b**) generated from the Cloud Compare software.

The results from Phase 1, where the frictional performances of slab sections were determined as SRT values, are shown in Table 4. In the first part of the results analysis, measured SRT values were qualitatively compared to the resulting height maps and histograms of the analyzed slab sections (Figure 4), to investigate whether the areas with generally higher texture values provide a higher SRT value. This initial comparison resulted in a conclusion that lower SRT values are not necessarily connected to the lower height values. For example, the average measured SRT value is the lowest for Section 1. By comparing the frictional performance of this section with height maps shown on Figure 4 left, it can be seen that the section with the lowest texture height values is Section 2 (it has the largest light-colored area). Furthermore, when comparing the point heights in Section 1 and Section 3 at the central section area, they are not corresponding to the SRT values measured. The central area where the SRT was measured in Section 3 has more points with lower height values (yellow- and green-colored points) than the central area in Section 1, while the SRT values are significantly different, and in favor of Section 3. However, the height map and histogram for Section 4 do show that it contains the most points with the largest height values, which corresponds to the highest SRT value measured.

The next step in the analysis was the surface data analysis in terms of texture parameters that can be calculated from the given dataset and compared to the measured friction expressed as SRT values. In order to compare the measured SRT values with texture properties, a smaller sub-section was extracted from each previously defined slab section. The sub-section was defined to be long and wide enough to cover the slab section area measured with the pendulum in the skid resistance test. At each extracted sub-section, five 2D profiles were generated along the sub-section at an equal distance covering the entire sub-section width. The profiles were extracted as coordinate-defined dense point clouds with XZ coordinates used for data analysis. The profile data were used to calculate the mean profile depth (MPD) and corresponding estimated texture depth (ETD) for each extracted profile. These two parameters are standard for pavement macro-texture performance assessment, and they are traditionally related to the frictional performance of pavement surfaces. Overall, 20 2D profiles were subjected to the texture parameters calculation. Acquired MPD and ETD values for all profiles in one sub-section were averaged to represent a unique texture indicator. Additionally, standard deviation was calculated for MPD values to inspect the homogeneity of the profiles in each sub-section. The results of profile data calculation and analysis are shown in Table 5, where it can be seen that the highest average MPD value is calculated for Section 3, while the lowest average value is calculated for Section 1. By analyzing the standard deviation of calculated MPD values for each section, it can be concluded that the most homogenous section is Section 2, while the section with the greatest MPD difference is Section 3. 

The next step was to inspect the possible correlation between the calculated MPD parameter acquired from the generated digital texture model and measured SRT values. As previously mentioned, the correlation between texture parameters acquired by standard measuring methods and corresponding frictional performance indicators of pavement surfaces is not always straightforward or significant enough. The idea was to investigate if the texture parameters obtained from an alternative texture acquisition method could yield a better correlation with measured friction parameters. Besides the standard texture characterization parameter MPD, five additional texture-related parameters were calculated from the digital pavement surface model according to the relevant regulation [3]. As the preliminary investigation was based on 2D profile analysis, the selected parameters were peak height R_m_, average height R_a_, root mean square roughness R_ms_, skewness R_sk_ and kurtosis R_ku_. These parameters are used for texture features characterization, regardless of the observed texture scale [4,49] and they were calculated for each measured sub-section on five extracted profiles, and averaged for the correlation analysis with the SRT values. In Table 6, the average values of all texture parameters calculated for the extracted profiles are shown. For each texture parameter, a Pearson correlation coefficient calculated for the measured SRT value is given.

All absolute values of Pearson correlation coefficients are higher than 0.7, which indicates that the correlation between calculated texture parameters and measured skid resistance is strong. MPD values show the best correlation with the SRT values having correlation coefficient 0.81. Correlation analysis resulted in negative correlation coefficient between measured frictional performance and calculated skewness and kurtosis values. According to [3], skewness values indicate the direction of profile peaks, where positive skew represents upwards directed peaks and negative skew represents the majority of profile peaks directed downwards. Therefore, a negative correlation coefficient leads to the conclusion that increases in skewness will reduce the SRT value. Kurtosis represents the “flatness” of a given distribution in comparison to normal distribution, where higher values indicate sharper distribution curves and heavy tails or outliers. As the statistical analysis resulted in negative correlation between SRT and R_ku_ values, it can be concluded that profiles with normal height distribution and lower kurtosis values would result in better frictional performance measured in SRT values. Further data analysis investigated the relation between all calculated alternative texture-related parameters and standard texture parameter MPD. A correlation analysis was performed for the parameters’ values calculated for all 20 extracted profiles and acquired correlation coefficients are given in Table 7. From the results, it can be seen that standard texture parameter MPD has a very strong correlation with R_m_ parameter and R_ms_ parameter correlates very strongly with both R_m_ and R_a_. Skewness and kurtosis values correlated negatively with all other texture parameters.

A multiple regression analysis was performed for measured skid resistance values and selected texture parameters with the highest correlation coefficients obtained: MPD, R_m,_ R_a_ and R_ms_. Resulting regression statistics are given in Table 8, where it can be seen that the multiple R and R^2^ values are high for analysis observing friction measurements SRT and MPD with R_m_ and R_ms_ alternative texture parameters. A moderate relation is obtained for the analysis performed on SRT values and three alternative parameters R_m,_ R_a_ and R_ms_. However, from the values of the remaining statistical parameters, it can be concluded that the acquired relations cannot be considered statistically significant, which could be addressed by a small number of observations.

The performed statistical analysis indicates that the frictional response of a pavement surface expressed as SRT value and texture parameters obtained from 3D digital texture model can be related, as the calculated correlation coefficients are considerably high for all inspected relations. Furthermore, SRT value can be an indicator for both the micro- and macro-texture effects on the frictional response of the pavement surface. Texture parameters were analyzed without separating the texture profile data according to the micro- or macro-texture level, and the correlation coefficient obtained between MPD and SRT is significant enough to conclude that also macro-texture influences frictional performance, even though low-speed skid resistance measurements are categorized as indirect methods for micro-texture determination. The results of a small-scale study indicate that the texture parameters derived from the digital texture model show better correlation with the frictional performance of the surface in comparison to the standard in situ measurements, as reported in [11]. However, further research on a larger set of data is needed to be able to describe the friction–texture relationship more thoroughly and with more statistical significance. The results of this research fit well with the conclusions from the research of the texture–friction relationship analyzed with similar photometric methods, showing that a more detailed texture analysis with additional calculated texture parameters could describe this phenomenon more precisely in comparison to traditional texture characterization methods [7,25,28,30,31].

## 5. Conclusions

Image analysis methods are novel methods of pavement texture data acquisition and characterization. By using different image analysis methods, it is possible to extract additional texture-related parameters that cannot be acquired by standard texture analysis methods. An extensive literature research was conducted to summarize the properties of different image analysis methods in terms of data acquisition and resulting texture parameters. From the performed literature review, some important conclusions are:

- Image analysis methods provide more detailed description of pavement texture properties than standard methods, enabling the extraction of texture parameters related to its geometrical, statistical and spectral characteristics in both 2D and 3D;
- The relationship between pavement texture and frictional response can be described much more precisely by using output parameters from image analysis methods;
- Data acquisition can be performed by using widely available equipment such as standard digital cameras, where the camera resolution governs the precision of a generated digital texture model;
- Image analysis methods do not provide a texture parameter right after the data acquisition, but demand some further data processing and analysis, which is a major limitation of the described methods.

In order to test the possibilities of advanced methods for pavement texture properties determination in terms of texture–friction relationship establishment, a new methodology for texture data analysis was established. The methodology represents an adjustment of previously applied methods for the digitalization of larger scale engineering problems and analysis in terms of dense point cloud data to a much smaller scale problem dealing with pavement surface texture. In comparison to similar performed research in this field, the developed methodology provides a more simple and accessible data acquisition procedure. It involves the application of an orthographic photogrammetry method for generating a set of digital texture images which are further processed in a commercial software for 3D mesh model creation and afterwards analyzed as a dense point cloud, with texture described as a set of points with XYZ coordinates. From the created dense point cloud of pavement texture, it is possible to extract sections and profiles which can be analyzed in terms of texture parameters significant for the frictional characteristics of the inspected surface. In order to test the developed methodology for 3D digital pavement texture model creation, a preliminary analysis was conducted, focusing on the calculation of the standard texture parameter MPD and five other texture descriptors. The calculated values of texture parameters were correlated to the measured friction performance expressed as SRT values. The results of correlation analysis showed that texture parameters calculated from the 3D model can be related to the friction performance of the surface, as the achieved correlation coefficients are higher than 0.7. This conclusion follows the conclusions from previously performed research that exploited advanced methods for pavement texture characterization, and achieved satisfactory relations with measured friction performance. However, the results from this study are only preliminary, as they were achieved for a very limited dataset. A much-detailed analysis of texture–friction relationship is planned for further research on a larger dataset, including various types of asphalt surfaces. The limitations of the developed methodology involve the problem of data acquisition precision, as no exact settings are defined prior to the image capturing procedure, but they depend on the operator’s knowledge and experience within this field of expertise. Moreover, experience is needed to ensure proper sharpness, brightness, and contrast settings, in order to acquire the best possible data with respect to the illumination and specific texture properties of the inspected surface. The precision of the acquired data could be improved by using multiple vision photometric methods or more sophisticated equipment. However, using a single digital camera makes this method accessible to both researchers and practitioners in the field.

The results of this preliminary study showed that the established methodology is applicable for the analysis of the pavement texture and friction relationship. By using the defined methodology for digital 3D texture model generation and acquired data analysis, a detailed investigation of additional profile-based and spatial parameters is planned for laboratory-produced asphalt slabs with different material properties, but also for the in situ measurement of constructed pavements. Furthermore, the development of a contact model for the prediction of pavement surface frictional response is planned as a next research step, with an emphasis on the influence of pavement texture properties resulting from the established digital texture model acquisition methodology.

## Figures and Tables

**Table 1:** Standard pavement macro-texture measuring methods and output data.

Acquisition Method	Measurement Method	Output Parameter
Contact	Sand patch test, Grease test, Outflow Meter	Mean Texture Depth (MTD), Estimated Texture Depth (ETD)
Non-contact	Sensor-based (laser) profilometers	Mean Profile Depth (MPD)

**Table 2:** An overview of image analysis methods for pavement texture characterization.

Data Acquisition Method	Resulting Entity	Output Parameters	Method Advantages	Method Limitations	References
Photometric methods (Stereo photogrammetry and Structure from Motion)	Digital 3D texture model from acquired images or laser scans: 3D mesh, 3D point cloud data or other XYZ-coordinate defined entities	Profile-based 2D parameters: MPD, peak radius, peak height, peak curvature, average roughness, peak to valley height, leveling depth, surface roughness depth, variance, average quadratic deviation, skewness and kurtosis Spatial parameters in 3D: amplitude parameters, spacing parameters, hybrid parameters and functional or feature parameters Texture parameters expressed as mathematical functions: Fast Fourier Transformation, Power Spectral Density, wavelets	More detailed representation of surface texture than standard texture measuring methods, fast and simple to use with basic knowledge of usage of photographic equipment	More complex and time consuming than the traditional methods for texture data acquisition; subjected to errors in the data acquisition procedure which can influence the accuracy of the data	[7,8,9,19,20,21,22,23,24,25]
Laser scanning			More detailed representation of surface texture than standard texture measuring methods, fast and reliable method less attributed to data acquisition errors in comparison to photography based methods	More expensive equipment in comparison to digital photography methods, more complicated for users (requires additional knowledge)	[4,5,10,15,16,21,26,27,28,29,30,31]
Single photography	Single surface photography (2D)	Mix design parameters (aggregate gradation, binder content, air voids volume) measured and analyzed from the binary images (optical analysis or edge detection technique) or cross-section images data	Simple acquisition method with basic output resulting images	Lack of pavement texture geometry knowledge important for frictional characteristics of the pavement surface	
	Cross-section photography (2D)				[32,33,34,35,36]

**Table 3:** Methodology for 3D texture model acquisition.

Procedure Step	Description	Result (Image)
Preparation of asphalt slab for data acquisition	Asphalt slab is brushed and cleaned to remove dust particles and debris; the surface is sprayed with anti-reflectance spray to reduce the effect of ambient light and minimize the image acquisition errors	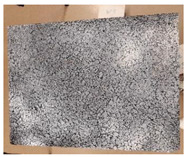
Image acquisition by orthographic photogrammetry method	Asphalt slab is placed on a fixed mount and lifted from its base for approx. 20 cm; digital camera is set on a mount 30 cm up and orthogonal to the slab surface; camera movement is left-right and up-down along the slab surface with no vertical movements; additional photos were taken along the slab edges at 45° to account for texture depth	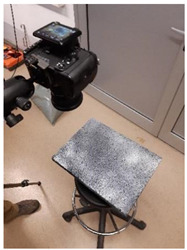
Image processing by Agisoft Metashape software	Acquired slab images are imported into software for 3D model generation; generated model is a 3D surface mesh with XYZ point coordinates	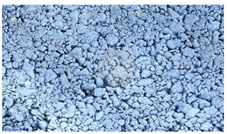
3D data analysis	3D mesh model is loaded in open-source software for point cloud data analysis Cloud Compare; 3D point cloud data model is created (upper photo); slab is divided into sections corresponding to the SRT-tested sections; from each section, several 2D profiles are extracted for calculation of relevant texture parameters (lower photo)	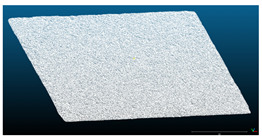 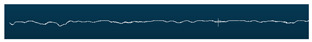
2D profile analysis	Extracted profiles are analyzed in terms of texture indicators: MPD and several alternative indicators	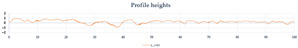

**Table 4:** Skid resistance measuring results.

Section No.	SRT Measurement					SRT (Average) [Unitless]
	No. 1	No. 2	No. 3	No. 4	No. 5	
I	75	75	76	77	76	75.8
II	79	77	79	79	77	78.2
III	84	84	83	84	83	83.6
IV	89	90	90	92	90	90.2

**Table 5:** MPD values calculated on extracted section segments.

Sub-Section No.	I	II	III	IV
MPD calculated [mm]	0.509	0.478	0.575	0.574
	0.379	0.454	0.509	0.597
	0.273	0.483	0.592	0.589
	0.299	0.447	0.762	0.531
	0.331	0.453	0.518	0.484
MPD averaged [mm]	0.358	0.463	0.591	0.555
ETD calculated [mm]	0.486	0.570	0.673	0.644
MPD standard deviation	0.093	0.016	0.102	0.047

**Table 6:** Correlation analysis of friction vs. texture values.

Section No.	SRT	MPD	R_m_	R_a_	R_ms_	R_sk_	R_ku_
1	75.8	0.358	1.392	0.288	0.3375	5.5261	14.3401
2	78.2	0.463	2.011	0.413	0.4870	2.9957	6.7700
3	83.6	0.591	2.512	0.668	0.7358	2.4662	4.9333
4	90.2	0.555	2.221	0.537	0.6153	2.3095	4.9706
Calculated correlation coefficient SRT vs. texture parameter		0.81	0.71	0.70	0.73	−0.77	−0.75

**Table 7:** Correlation coefficients between calculated texture parameters.

Calculated Texture Parameter	MPD	R_m_	R_a_	R_ms_	R_sk_	R_ku_
MPD	1	0.8190	0.7274	0.7463	−0.7802	−0.6869
R_m_	0.8190	1	0.9705	0.9784	−0.7879	−0.7070
R_a_	0.7274	0.9705	1	0.9989	−0.6953	−0.6235
R_ms_	0.7463	0.9784	0.9989	1	−0.7186	−0.6447
R_sk_	−0.7802	−0.7879	−0.6953	−0.7186	1	0.9814
R_ku_	−0.6869	−0.7070	−0.6235	−0.6447	0.9814	1

**Table 8:** Regression analysis of friction vs. texture parameters.

Friction vs. Texture Parameters	Regression Analysis Results				
	Multiple R Value	R^2^ Value	F Value	Significance F	*p*-Values (Intercept; Variable 1; Variable 2)
SRT, MPD, R_m_	0.955	0.9121	5.1879	0.2965	0.1203; 0.2720; 0.335
SRT, MPD, R_ms_	0.9252	0.8560	2.9731	0.3794	0.4612; 0.3733; 0.4444
SRT, R_ms_, R_m_	0.7321	0.5359	0.5774	0.6812	0.2551; 0.8024; 0.9278
SRT, R_ms_, R_a_	0.8021	0.6433	0.9018	0.5972	0.2104; 0.6385; 0.6732

## Data Availability

Data collected through this research and presented in the paper are available on request from the corresponding author. Data are not publicly available because they are part of an ongoing research for doctoral thesis.
